# Exploiting non-permissive CHO cells as a rapid and efficient method for recombinant HSV-1 isolation

**DOI:** 10.1186/s13568-024-01709-0

**Published:** 2024-05-09

**Authors:** Mishar Kelishadi, Hosein Shahsavarani, Alijan Tabarraei, Mohammad Ali Shokrgozar, Amirabbas Rahimi, Ladan Teimoori-Toolabi, Kayhan Azadmanesh

**Affiliations:** 1 Department of Molecular Virology, Pasture Institute of Iran, Tehran, Iran; 2 Department of Cell and Molecular Biology, Faculty of Life Science and Biotechnology, Shahid Beheshti University, Tehran, Iran; 3 Laboratory of Regenerative Medicine and Biomedical Innovations, Pasteur Institute of Iran, National Cell Bank, Tehran, Iran; 4 The Iranian Biological Resources Center, Academic Center for Education, Culture and Research (ACECR), Tehran, Iran; 5 Infectious Diseases Research Center, Golestan University of Medical Sciences, Gorgan, Iran; 6 Department of Virology, Faculty of Medicine, Golestan University of Medical Sciences, Gorgan, Iran; 7 Molecular Medicine Department, Biotechnology Research Center, Pasteur Institute of Iran, Tehran, Iran

**Keywords:** Fluorescent reporter, CHO cells, Oncolytic HSV-1, Purification plaque, BHK-21, Non-permissive

## Abstract

Using herpes simplex virus type 1 (HSV-1) as a therapeutic tool has recently emerged as a promising strategy for enhancing the treatment of various cancers, particularly those associated with the nervous system, which is the virus's natural site of infection. These viruses are specifically engineered to infect and eradicate tumor cells while leaving healthy cells unharmed. To introduce targeted mutations in specific viral genes, gene-modification techniques such as shuttle vector homologous recombination are commonly employed. Plaque purification is then utilized to select and purify the recombinant virus from the parental viruses. However, plaque purification becomes problematic when the insertion of the desired gene at the target site hampers progeny virus replication, resulting in a lower titer of cell-released virus than the parental virus. This necessitates a laborious initial screening process using approximately 10–15 tissue culture dishes (10 cm), making plaque purification time-consuming and demanding. Although the recently developed CRISPR-Cas9 system significantly enhances the efficiency of homologous integration and editing precision in viral genes, the purification of recombinant variants remains a tedious task. In this study, we propose a rapid and innovative method that employs non-permissive Chinese hamster ovary (CHO) cells, representing a remarkable improvement over the aforementioned arduous process. With this approach, only 1–2 rounds of plaque purification are required. Our proposed protocol demonstrates great potential as a viable alternative to current methods for isolating and purifying recombinant HSV-1 variants expressing fluorescent reporter genes using CHO cells and plaque assays.

## Introduction

HSV-1(Herpes simplex virus type 1), also known as human alpha herpes virus 1, is a common eukaryotic pathogen belonging to the Herpesviridae family. It possesses a relatively large genome of approximately 152 kilobases, consisting of linear double-stranded DNA. This genome encodes around 80 proteins, and interestingly, nearly half of these proteins are not essential for virus replication (Conner et al. 2005; Roehm et al. 2016; Oh et al. 2019).

Recombinant HSV-1 has emerged as a novel platform for the development of tumor-targeted replicating oncolytic therapies. Due to the absence of integration into the human genome, the viral genome offers several advantageous features such as foreign gene cloning, efficient replication, broad cell host range, and enhanced safety (Lin et al. 2016). These viruses are particularly valuable in fighting nervous system-related cancers due to their natural infection site. At present, diverse oncolytic HSV-1 variants have displayed promising outcomes in clinical trials (Fu et al. 2001, Varghese et al. 2002, Gianni et al. 2004; Kagabu et al. 2021).

Nevertheless, shuttle vector homologous recombination stands as a conventional technique for HSV-1 genome engineering. The shuttle vector carries a homologous sequence that undergoes recombination with HSV-1 within pre-infected cells (Russell et al. 2015; Lin et al. 2016, Lee et al. 2019).

The utilization of the recently developed CRISPR-Cas9 system has shown significant improvements in homologous integration efficiency and editing precision of viral genes. Originally identified as a bacterial anti-viral defense mechanism, this powerful and dependable system has been instrumental in the creation of antiviral agents, oncolytic viruses, and vaccines (Suenaga et al. 2014; Yuan et al. 2016). Presently, ongoing studies indicate that despite the substantial advancements in genome editing and gene replacement facilitated by CRISPR/Cas9, there are still challenges associated with the isolation and purification of recombinant HSV-1 (Walker et al. 1977, Russell et al. 2015; Yuan et al. 2016; Li et al. 2018).

The use of fluorescent reporter genes-expressing shuttle vectors such as GFP (Liu et al. 2003)(green fluorescent protein), RFP (red fluorescent proteins) (Ramachandran et al. 2008), mCherry (Etienne et al. 2017) and YFP (yellow fluorescent protein) (Miyagawa et al. 2015) has become a prevalent strategy for effectively screening recombinant HSV-1 that expresses these specific fluorescent proteins. (Abdoli et al. 2017; Scanlan et al. 2022). These viruses can be readily identified through fluorescent microscopy, allowing for easy detection. Moreover, they can be isolated from other viruses using the plaque purification method. Due to these capabilities, they serve as a fundamental platform for developing advanced generations of oncolytic HSV (oHSV), designing vaccines, and implementing cancer therapy approaches (Walker et al. 1977, Russell et al. 2015; Yuan et al. 2016). Nevertheless, plaque purification posses challenges when the insertion of a desired gene at the targeted site hampers progeny virus replication, resulting in lower titers of cell-released virus compared to the parental virus. Typically, around 15–10 tissue culture dishes (10 cm) are needed, making the initial screening process arduous and time-consuming. Consequently, there is a pressing need for advancements in the screening procedure to streamline and expedite the plaque purification process.

Chinese hamster ovary (CHO) cells are prominently employed as a key cell line in the manufacturing of biopharmaceuticals and recombinant proteins (Bačnik et al. 2021). Due to the ability to generate high protein yields, a well-established safety profile of products and the convenience of suspension culture in a serum-free environment (as serum can be a potential source of contamination by viruses, mycoplasmas, and even prions), CHO cells have become a preferred choice for the production of biopharmaceuticals and recombinant proteins (Berting et al. 2010). Despite their widespread use in biopharmaceutical production, it is important to be aware that CHO cells have the potential to be infected by various types of viruses. These can include Adenovirus, Vesicular stomatitis virus, Reovirus, Parainfluenza virus, Bovine respiratory syncytial virus, and Bluetongue virus (Berting et al. 2010). However, they are indeed less sensitive to infection by other viruses compared to other cell lines used in the production of recombinant proteins, such as baby hamster kidney (BHK) cells (Li et al. 2019). However, CHO cells and are naturally resistant to HSV-1 infection (Nicola et al. 2004).

Here, we present a breakthrough discovery highlighting the susceptibility of CHO cells to HSV-1 infection. We introduce a novel and rapid method utilizing CHO cells, which represents a significant improvement over the previously laborious process. Our approach offers a practical screening option to overcome these challenges and streamline the isolation and purification of desired recombinant viruses. By employing non-permissive CHO cells, we achieve a remarkable enhancement in efficiency. With this method, only 1–2 rounds of plaque purification may be required, expediting the overall process. Moreover, we focused on the manipulation of the immediate-early gene US12, which encodes polypeptide 47 (ICP47) in the context of ICP34.5-deleted HSV-1. As an illustrative example, the study describes the insertion of an EGFP expression cassette into the US12 gene using homologous recombination. The ICP34.5 protein plays crucial roles in viral replication, viral exit from infected cells, prevention of premature shut-off of protein synthesis in the infected host, and neurovirulence. Normally, ICP47 functions by inhibiting antigen presentation in HSV-infected cells. However, disrupting ICP47 results in a virus that fails to confer properties on infected tumor cells that would shield them from the host's immune system upon HSV infection. This product holds promise for oncolytic virotherapy research and the creation of further recombinant HSV constructs (Liu et al. 2003).

## Materials and methods

### Cell lines

In this study, three different cell lines were utilized: Vero cells (African green monkey kidney, NCBI-C101*)*, BHK-21 cells (Baby hamster kidney, NCBI-C107*),* and CHO-K1 cells (Chinese hamster ovary; NCBI-C644). These cell lines were sourced from the National Cell Bank of Pasteur Institute of Iran.

The cells were cultured in high glucose Dulbecco’s modified Eagle’s medium (DMEM) (Gibco, Germany) supplemented with 10% heat-inactivated fetal bovine serum (FBS; Gibco), 2 mM l-glutamine and 1% penicillin/streptomycin (Gibco, Germany) at 37 °C with a humidified atmosphere containing 5% CO2. It is important to note that all cell lines used in the study were confirmed to be free from contaminations.

### Viruses

The principal virus used in this study was Δγ34.5/HSV-1 (Red). This virus was derived from an ICP34.5-null mutant of HSV-1, where both copies of ICP34.5 contained a BleCherry reporter gene driven by the cytomegalovirus promoter. The Δγ34.5/HSV-1 served as a reporter virus for identifying and verifying homologous recombination (Abdoli et al. 2017; Haghighi-Najafabadi et al. 2021).

The construction of Δγ34.5/HSV-1 was achieved using a standard homologous recombination-based method, starting from HSV-1 obtained from Dr. Houriyeh Soleimanjahi of Tarbiat Modarres University, Tehran, Iran. The virus was available at the Virology Department of Pasteur Institute of Iran (Abdoli et al. 2017; Haghighi-Najafabadi et al. 2021).

For viral propagation, Vero cells were cultured in DMEM media supplemented with 10% FBS and 1% penicillin/streptomycin. The cells were maintained at 37 °C with 5% CO2 in a humidified atmosphere. After observing cytopathic effects within 24–72 h, the cells were harvested, and subjected to freeze–thaw cycles, and the viral supernatant was collected. The supernatant was then filtered through 0.45 µm pores, tittered using a plaque assay, aliquoted, and stored at −80 °C for further use.

### Infection of BHK cells with Δγ34.5/HSV-1

In the initial step, BHK cells were infected with Δγ34.5/HSV-1 a multiplicity of infection (MOI) of 1. The cells were harvested after 48 h cytopathic effects (CPE) resulting from viral replication were observed, Following the observation of cytopathic effects, the viral supernatant was filtered, divided into smaller aliquots, tittered, and subsequently stored at −80 °C.

### DNA extraction

DNA was extracted from the stock of virus-infected BHK cells using the High Pure Extraction Kit (Roche Diagnostics GmbH, Mannheim, Germany), following the instructions provided by the manufacturer.

### ICP47 shuttle vector construction for homologous recombination (HR)

The ICP47 shuttle vector used in the present study was constructed by Haghighi-Najafabadi et al. and was obtained from the Virology Department of the Pasteur Institute of Iran. The construction process involved amplifying two fragments, one upstream and one downstream, each encoding specific regions of the genome. These fragments were of lengths 1638 and 1502 nucleotides, respectively. Additionally, the vector contained the US12 CDS of HSV-1, an EGFP expression cassette controlled by the CMV promoter, and a BGH terminator. The fragments were subcloned into pSL1180, resulting in the generation of pSL1180-ARM1&2-ICP47-EGFP (Haghighi-Najafabadi et al. 2021).

### Generation of Δ47/Δγ34.5 HSV-1 through homologous recombination

Transient transfections were performed on BHK cells using ScreenFect^™^ A plus—Fujifilm WAKO Corp. to following the manufacturer’s protocol. The optimal reagent: DNA ratio of 2:1 was used with 1 µg of DNA per well in a 24-well plate.

BHK cells were seeded in 24-well plates (SPL Life Sciences, Korea) at a density of 0.05 × 106 cells/well to achieve 80% confluency before transfection. The cells were transfected with the pSL1180-ARM1&2-ICP47-EGFP shuttle vector plasmid containing the homologous sequence. Following transfection, the cells were incubated at 37 °C with 5% CO2 for 24 h to allow for gene expression. On the following day, the same transfection procedure was repeated, with the addition of Δγ34.5/HSV-1 (at an MOI of 1) to the lipoplex complex. The resulting complexes (shuttle vector + virus) were then added to the same BHK cell monolayers cultured in 24-well plates with 0.35 mL complete medium. The BHK cell culture, containing the infection/transfection mixture, was harvested at 24–48 h when an 80% cytopathic effect was observed in the cell monolayer. The cells underwent three freeze–thaw cycles to release the viral particles, and the resulting samples were stored in aliquots at −80 °C for further use.

### Isolation and purification of mutant viruses by plaque purification

For the isolation of mutant viruses, Plaque purification was performed to isolate the mutant viruses*.* Briefly, Vero cells were seeded into 6-well plates; they were infected with different dilutions of the mutant viral supernatant. After 1 h incubation at 37 °C, the media was carefully aspirated, and the cells were overlaid with DMEM medium containing 1.5*%* carboxymethyl cellulose and incubated at 37 °C for 72 h to allow plaque formation. Daily monitoring of the plates was conducted using an inverted fluorescent microscope (Nikon Eclipse Ti-S, Japan) to observe EGFP expression. Plaques exhibiting EGFP expression indicated cells infected with the mutant viruses. Plaque purification was repeated multiple times to separate the mutant viruses from the parental viruses. This process ensured the isolation of individual mutant viral clones.

### Isolation and expansion of recombinant virus using non-permissive (CHO) cells (two rounds)

The aliquot vials suspected to contain mutated viruses were subjected to serial two-fold dilutions. Each dilution was then mixed with CHO cells at a density of 10^4^ cells per well and seeded in a 96-well tissue culture plate. The plate was observed daily using an inverted fluorescent microscope to detect the expression of EGFP, beginning from 1 to 3 days after infection.The wells that exhibited EGFP expression were treated with trypsin to detach the cells. Subsequently, the detached cells were transferred into a 100-mm cell culture dish that had been pre-seeded with CHO cells at a density of 5 × 105 cells per dish. The purpose of this transfer was to increase the spatial separation between the fluorescent-positive cells, allowing for individual clonal expansion and further analysis.

The individual cells that exhibited positive signals for both BleCherry and GFP (indicating the presence of the recombinant green/red virus) were carefully selected. These selected cells were detached using a cell scraper and subsequently propagated in BHK cells (or Vero cells). To release the viral particles, three freeze–thaw cycles were performed. The resulting virus, designated as Δ47 (GFP) /Δγ34.5 (BleCherry) HSV-1, was considered the mutant virus of interest. The mutant virus stock was divided into aliquots and underwent three additional freeze–thaw cycles to ensure proper preservation. The aliquots were then stored at −80 °C for future use.

### PCR analysis and sequencing for verification of homologous recombination

To verify the recombination process, the mutant virus DNA was extracted with High Pure Extraction Kit) Roche Diagnostics GmbH, Mannheim, Germany). PCR was then performed using a PCR master mix kit (Taq DNA Polymerase Master Mix RED 2x, Ampliqon, Denmark) The PCR reaction was carried out in a total volume of 25 containing 12.5 μl Taq DNA Polymerase, 1 × Master Mix RED, approximately (_~_100–150 ng) of HSV-1 DNA and 0.2 μM of each forward and reverse test primers listed in Table 1.

**Table 1:** Primer sequences for generating homologous fragments of US12 and recombination analysis

Primer	Sequence
Δ47/Δ34.5 HSV-1 construction	
Upstream homologous arm US12 forward	TCTAGAGGGTTCGATTGGCAATGTTGTCTCCC
Upstream homologous arm US12 reverse	GAGTCCCGGGTACGACCATCACCCG
Downstream homologous arm US12 forward	GCAAGCTTGCTCCCCCCCGACGAGCAGGAAG
Downstream homologous arm US12 reverse	GCATCGATCTTGTTCTCCGACGCCATC
Recombination analysis	
US12 test primer forward	GGTTGGGTCTGGCTCATCTC
US12 test primer reverse	CCCACCCACGAAACACAG

The PCR reaction was programmed as follows: initial denaturation at95 °C for 5 min; followed by 30 cycles of 95 °C for 60 s, 55 °C for 30 s, 72 °C for 2 min; a final extension step was performed at 72 °C for 5 min. The resulting PCR products were loaded onto a 1% agarose gel and visualized by exposing to ultraviolet (UV) light (1-kb DNA ladder, CinnaGen) (Haghighi-Najafabadi et al. 2021).

## Result

### Construction of Δ47/Δγ34.5 HSV-1

**Fig. 1:**
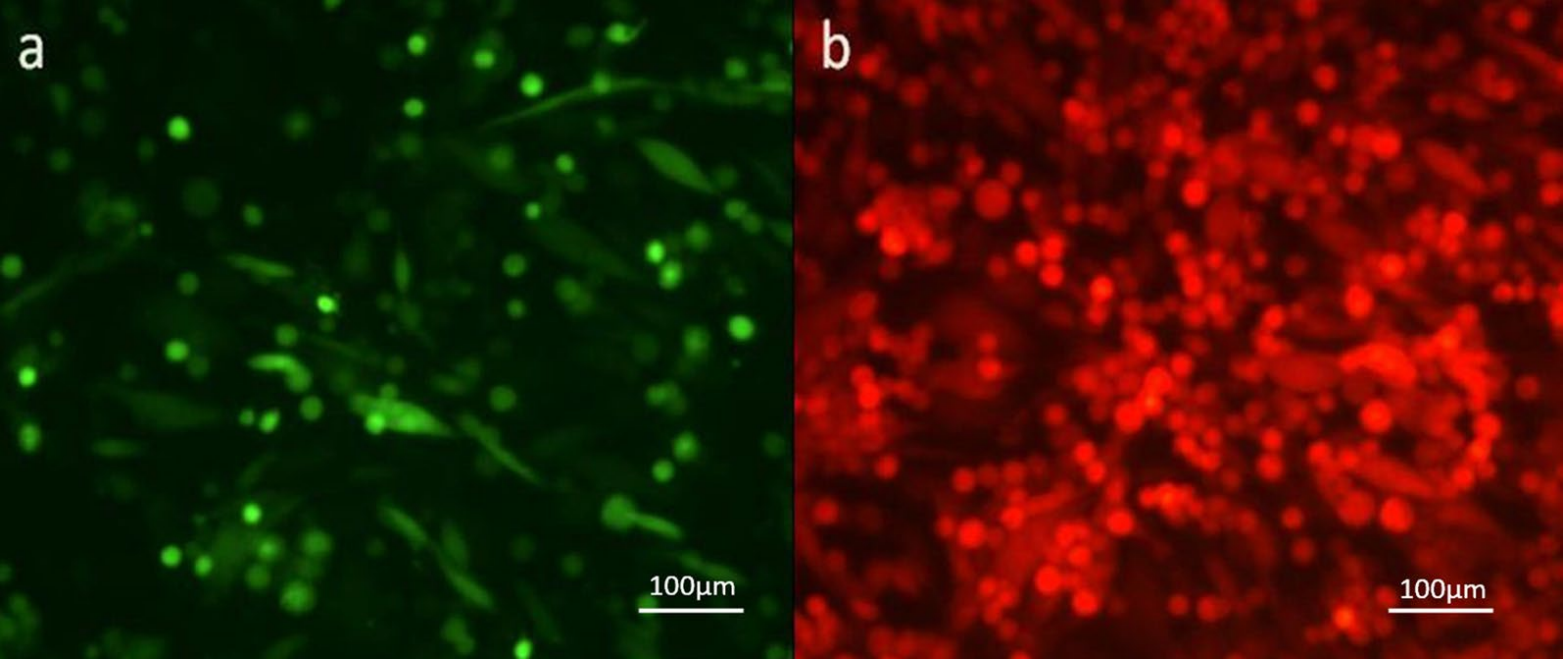
Imaging of the BHK cells one day after transfection/infection **a** Fluorescent microscopy analysis of transfection of pSL1180-ARM1&2-ICP47-EGFP plasmid expressing EGFP (magnification × 200) **b** Fluorescent microscopy analysis of Δγ34.5/HSV-1-BleCherry-positive infected cells expressing BleCherry (magnification × 200)

To construct a Δ47/Δ34.5 HSV-1, in the first step, BHK cells were transfected with a vector containing the pSL1180-ARM1&2-ICP47-EGFP fragment using a transfection method. To improve transfection efficiency, the same transfection procedure was repeated on the following day. The transfection efficiency was qualified by fluorescent microscopy verifying the expression of the EGFP gene, as illustrated in Fig. 1. In the second step, transfected BHK cells were infected with HSV-1/Δ34.5/Blecherry to construct a Δ47/Δ34.5 HSV-1.

### Isolation of Δ47/Δγ34.5 HSV-1 in Vero cell line

**Fig. 2:**
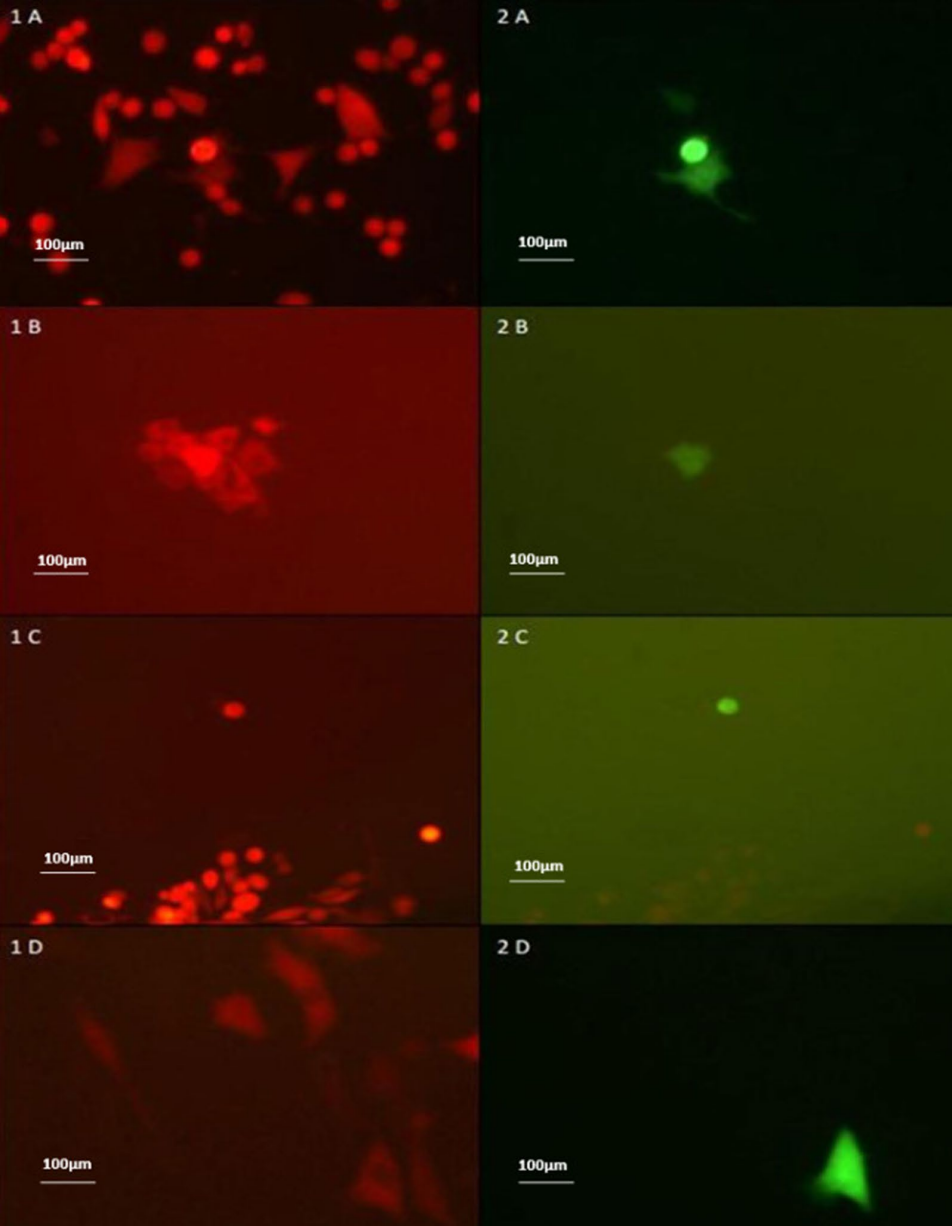
Challenges in screening procedure of recombinant HSV-1 in permissive cell lines (Exemplified in Figs. **A**, **B**, **C** and **D**)

To isolate the recombinant virus, plaque purification was performed in Vero cell line. As shown in Fig. 2, the plaque purification process can be problematic when the percentage of HSV-1 recombinants of interest be too low in compared with parental virus (10^–2^–2%, depend on the conventional homologous recombination method or CRISPR-Cas9 genome editing).

### Isolation of Δ47/Δγ34.5 HSV-1 in CHO cell line

**Fig. 3:**
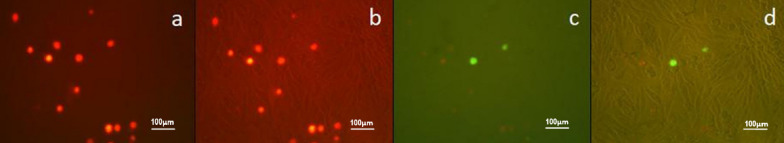
A blend of single cells containing the parent green Δ34.5 HSV-1 or recombinant Δ47 (green) /Δ34.5 (red) HSV-1; **a** Green excitation filter for BleCherry detection vision **b** Light + Green excitation filter for BleCherry detection vision **c** Blue excitation filter for EGFP detection vision **d** Light + Blue excitation filter for EGFP detection vision (Magnification × 200)

To improve the aforementioned arduous process, the transfection/infection supernatant harvest was mixed with CHO cells after three freeze–thaw cycles and seeded in 96-well tissue culture plates and monitored daily for EGFP expression. Fluorescent imaging was done at 1–3 days after infection. As shown in Fig. 3, the virus acquired from CHO cells is unable to infect CHO or spread to adjacent cells and infection is limited to a single cell of replication in CHO. So, EGFP-expressing cells are easily isolated.

**Fig. 4:**
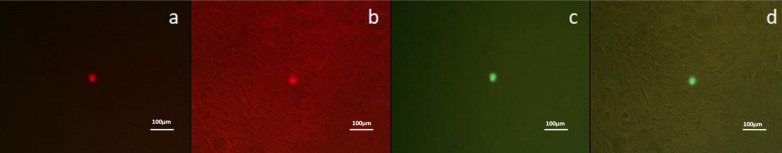
Fluorescent microscopy analysis of the single cell containing Δ47 (green)/Δ134.5 (red) HSV-1 after transferring into 100-mm cell culture dish pre-seeded with CHO cells; **a** UV vision (Green excitation filter for Blecherry) **b** Light vision + UV vision (Green excitation filter for Blecherry) **c** UV vision **d** Light vision + UV vision (Green excitation filter for Blecherry) (magnification × 200)

The wells harboring cells expressing EGFP were transferred to a 100-mm cell culture dish that had been preseeded with CHO cells, as illustrated in Fig. 4. This transfer was performed to provide a larger growth area for the EGFP-expressing cells and enable their continued propagation and analysis.

**Fig. 5:**
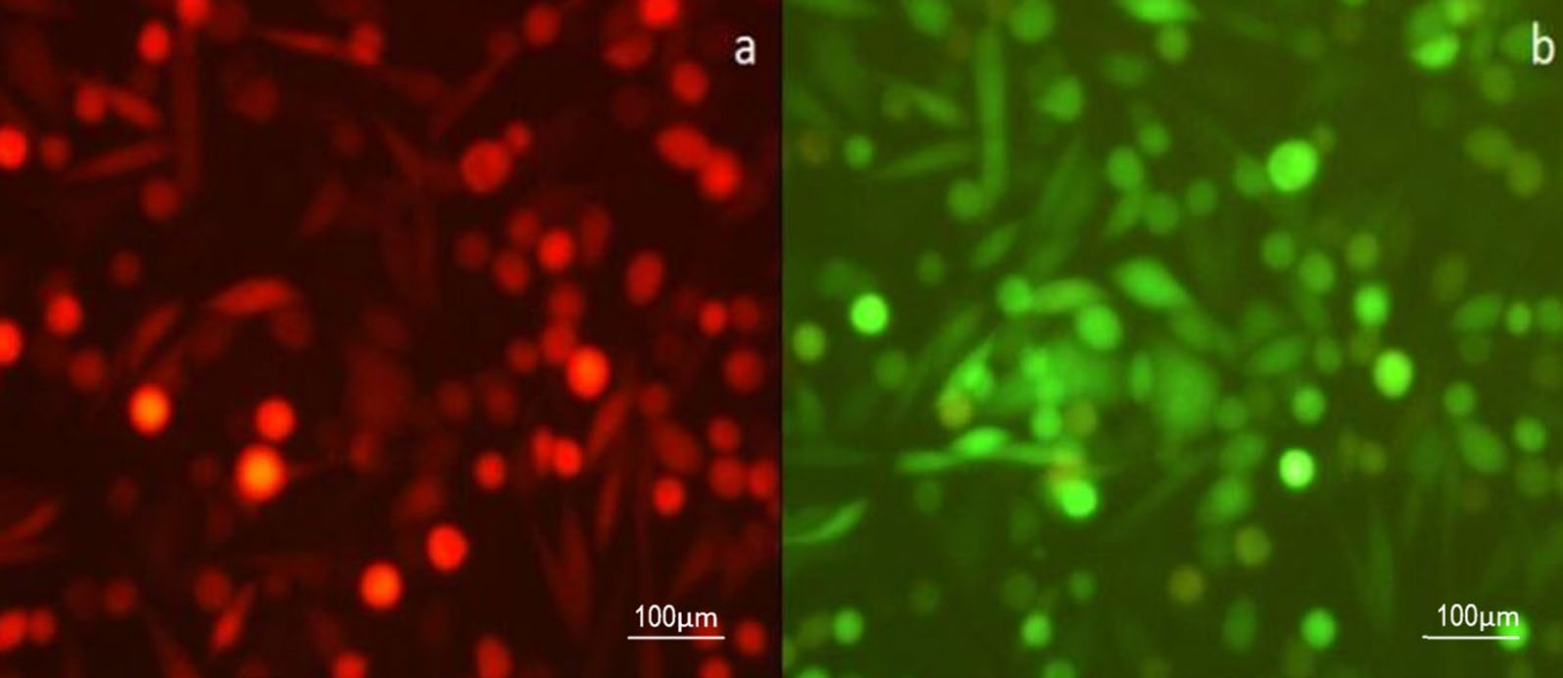
Expansion of the same single cell in BHK cell line. Fluorescent analysis of green/red Δ47/Δ134.5 HSV-1 by: **a** Green excitation filter for Blecherry detection **b** Blue excitation filter for Blecherry detection

The individual cells that exhibited positive signals for both BleCherry and GFP were cultured and propagated in BHK cells, as depicted in Fig. 5. To confirm the presence of the desired genetic modification, PCR analysis was performed. As anticipated, the PCR product obtained from the mutant virus showed an increased size at the ICP47 loci compared to the PCR product from the Δγ34.5/HSV-1 parental virus. This validated the presence of the EGFP expression cassette in the eliminated region of the viral genome. (Haghighi-Najafabadi et al. 2021).

## Discussion

In this study, we manipulated HSV-1 by replacing the US12 (ICP47) gene with the green fluorescence protein (GFP) in the Δγ34.5/HSV-1 virus. This genetic modification aimed to generate a replication-conditional mutant of HSV-1.

Our findings provide novel insights into the use of CHO cells for constructing and purifying Δ47/Δγ34.5 HSV-1 (As a representative of a fluorescent reporter HSV*‐*1), which circumvents certain challenges associated with conventional methods.

CHO-K1 cells are typically considered nonpermissive to HSV-1 infection due to their lack of gD receptors, making them resistant to HSV infections. (Brown et al. 1994, Roller et al. 1997, Conner et al. 2005). However, previous studies have shown that the ability of HSV-1 to infect CHO cells can be influenced by the cell type used for virus propagation, suggesting that cell and receptor characteristics play a significant role in the infection process (Brown et al. 1994; Gianni et al. 2004; Conner et al. 2005).

In our study, we initially investigated the infectability of CHO cells with Δγ34.5/HSV-1 expressing the BleCherry reporter gene. We successfully modified the US12 gene of Δγ34.5/HSV-1 using conventional homologous recombination. The results of BleCherry expression and titration showed that Δγ34.5/HSV-1 propagated on BHK cells (Δγ34.5/HSV-1/BHK) but not on Vero cells (Δγ34.5/HSV-1/Vero) was capable of infecting CHO cells, and productive progeny virions were produced.

To optimize the transfection efficiency, we found that double transfection of the cells significantly increased the transfection efficiency. Adding Δγ34.5/HSV-1 to the complex after lipoplex formation during the second transfection greatly enhanced the efficiency. Various virus doses should be tested to determine the optimal transfection process (Fig. 1) (Ishikawa et al. 1992, Fu et al. 2001, Burnham et al. 2016).

The infected CHO cells exhibited vulnerability to Δγ34.5/HSV-1 infection, with approximately 30–40% of exponentially growing CHO cells being infected by Δγ34.5/HSV-1 propagated from BHK cells. The simultaneous introduction of serum and virus after serum starvation of CHO cells for 24 h increased the infection rate to approximately 70% of cells. The vulnerability of CHO cells to HSV-1 infection appeared to depend on virus titers and the cell cycle status (Conner et al. 2005).

**Fig. 6:**
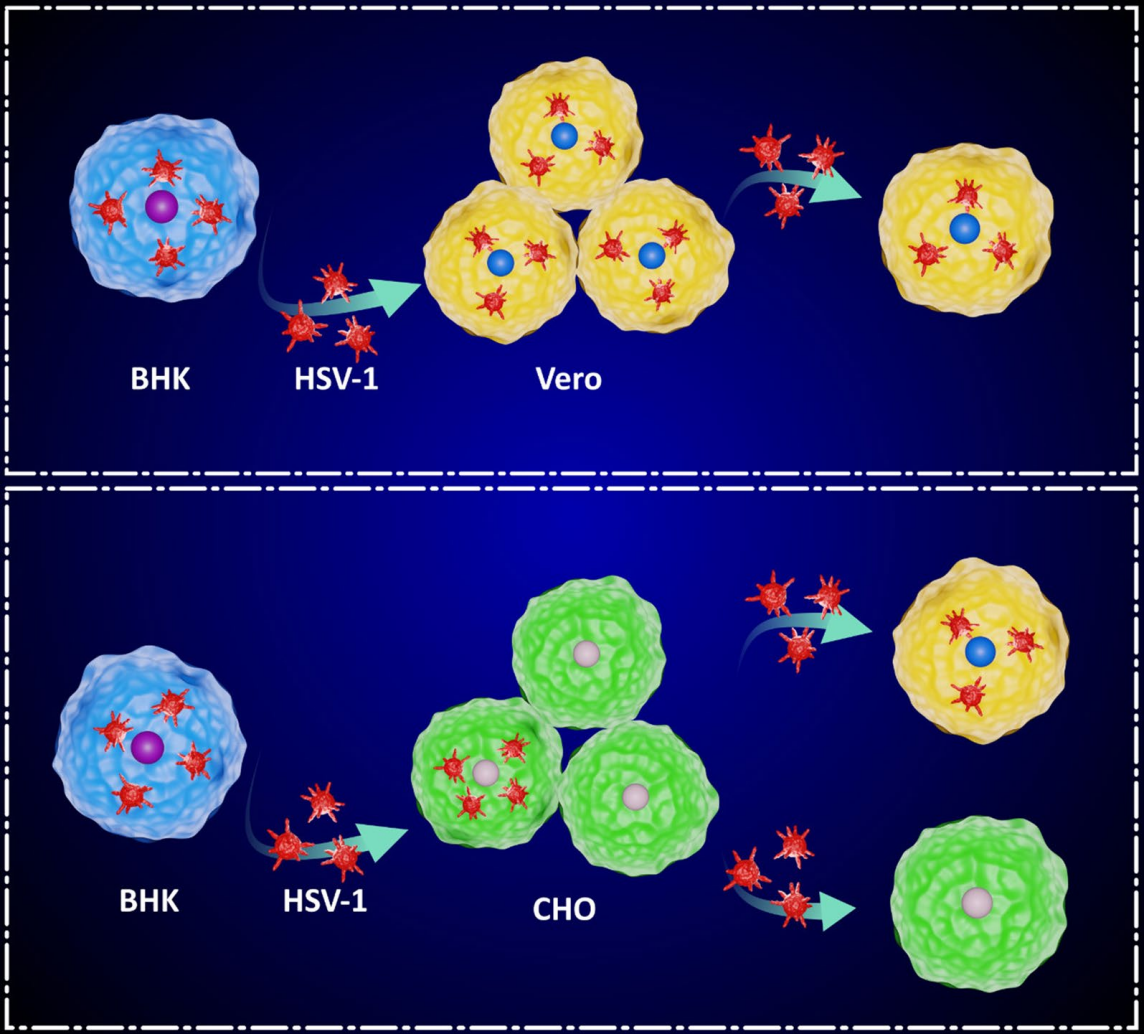
Schematic illustration of the Characteristics of CHO cell; the virus acquired from CHO cells is unable to infect CHO or spread to adjacent cells and infection is limited to a single round of replication in CHO cells whereas this ability is obtained in Vero cells

We observed that the infectious virions released into the supernatants of Δγ34.5/HSV-1/BHK-CHO infected cells were present at low levels, making them difficult to detect (Conner et al. 2005; Berting et al. 2010). However, when cells and supernatants were harvested together, the progeny viruses became readily detectable. This suggests that CHO cells do not efficiently release progeny viruses compared to BHK or Vero cells. Furthermore, the released virus from CHO cells exhibited limited infectivity and was unable to infect CHO cells or spread to adjacent cells. In contrast, BHK or Vero cells demonstrated the ability for multiple rounds of replication and cell-to-cell spread (Fig. 6) (Roller et al. 1997; Conner et al. 2005; Berting et al. 2010).

**Fig. 7:**
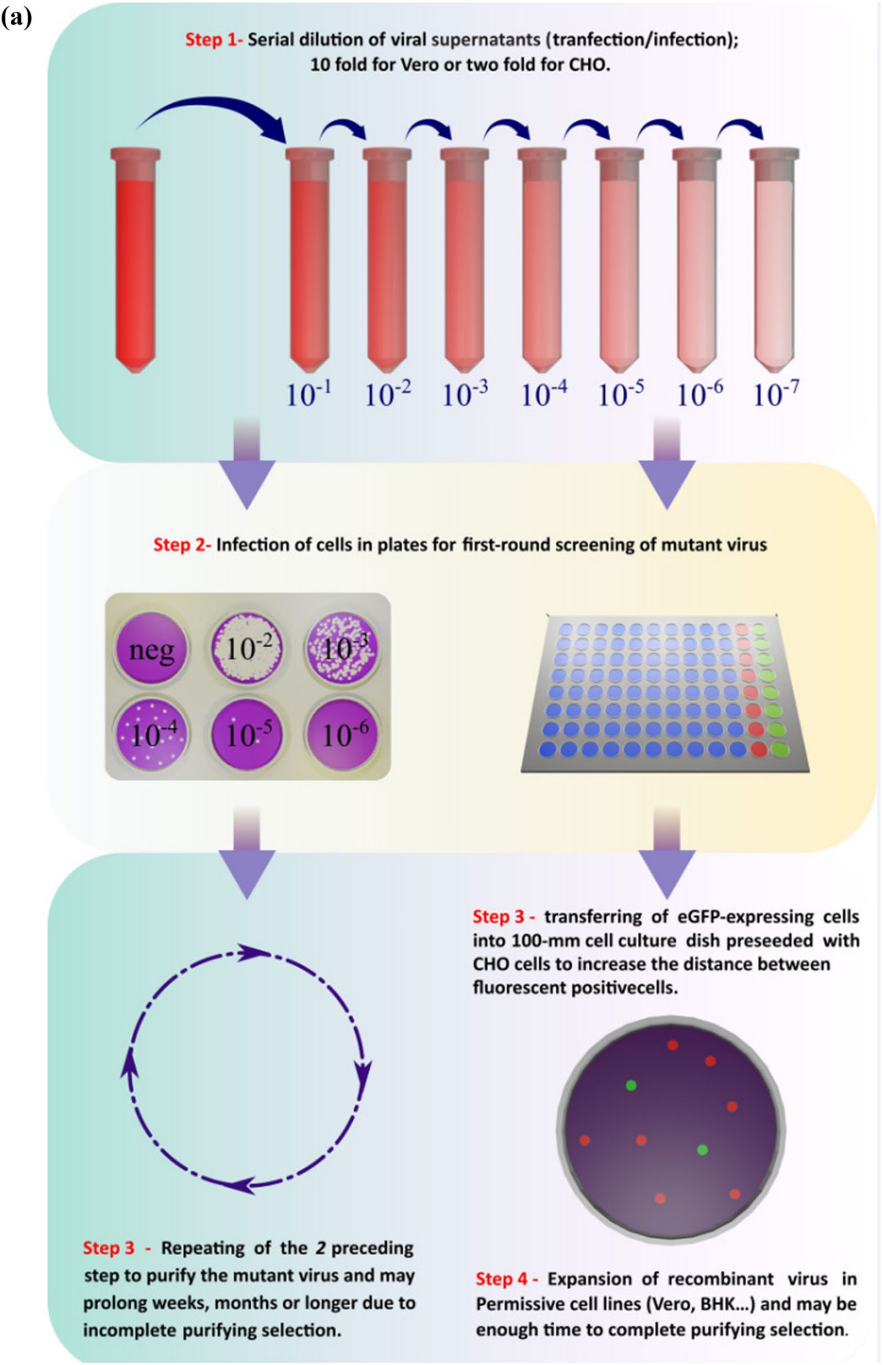

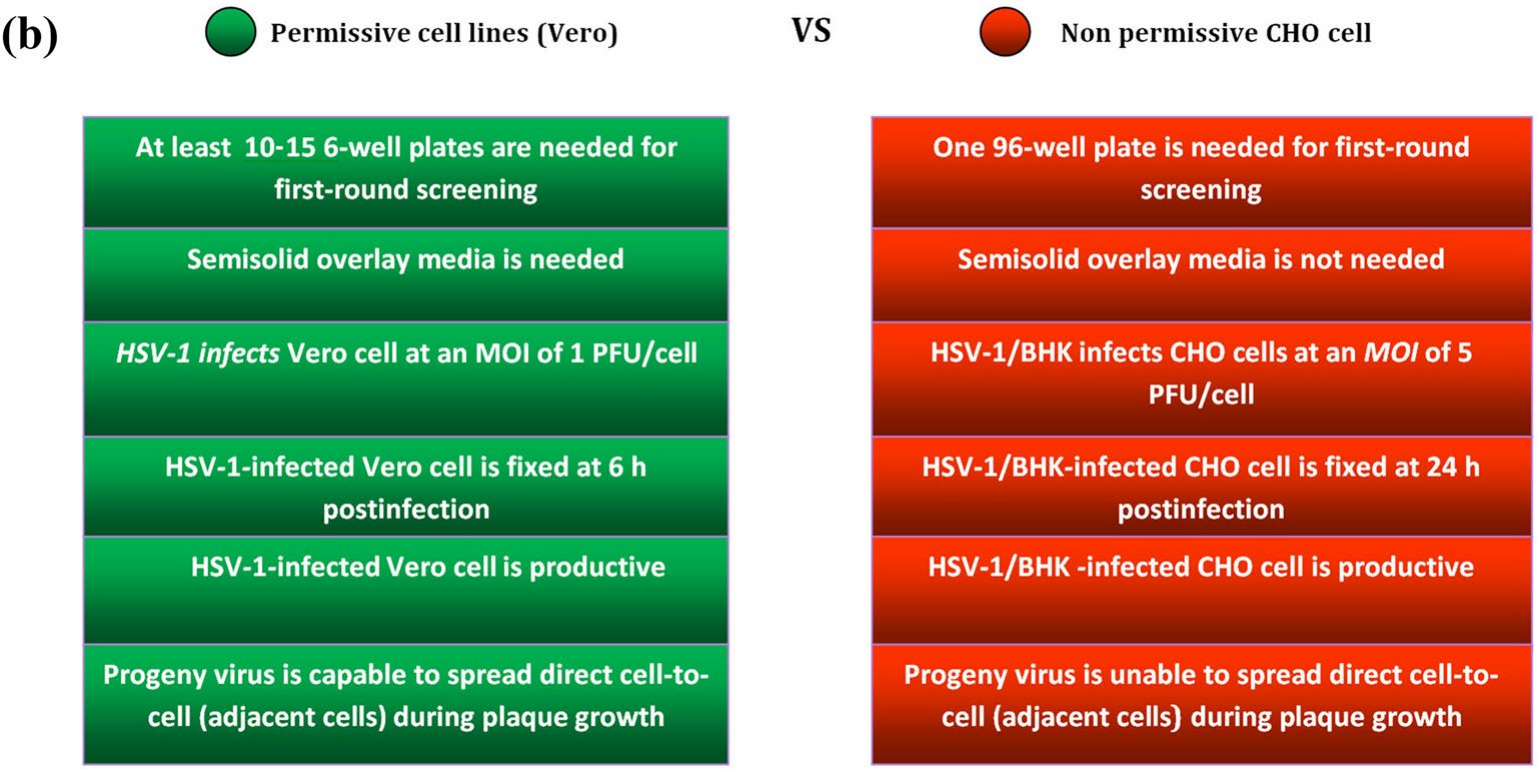
**a** Graphical protocol overview of two protocols for isolation and purification of recombinant HSV-1. **b** Characteristics and comparison of the two protocols

The use of CHO cells in this protocol offers several advantages. The need for semisolid overlay substrates, such as agarose or carboxymethyl cellulose, to prevent indiscriminate infection through the liquid growth medium, as required in the plaque purification technique, is eliminated. This makes the CHO system more cost-effective and overcomes the challenges associated with single plaque pick-up through the agarose layer (Fig. 7).

Our protocol successfully demonstrated the use of CHO cells as a viable alternative to the traditional plaque assay for the isolation and purification of recombinant HSV-1.

In conclusion, our study highlights the successful manipulation of HSV-1 using CHO cells as a potent platform for constructing and purifying recombinant viruses. This approach offers improved efficiency and cost-effectiveness compared to traditional approaches, making it a valuable tool for future research and applications involving HSV-1.

## Data Availability

The data used to support the fndings of this study are included within the article and the nucleotide sequence data are available in the GenBank databases.
